# Development of Loop-Mediated Isothermal Amplification Assay for Detection of Clinically Significant Members of *Acinetobacter calcoaceticus–baumannii* Complex and Associated Carbapenem Resistance

**DOI:** 10.3389/fmolb.2021.659256

**Published:** 2021-06-23

**Authors:** Amit Sharma, Rajni Gaind

**Affiliations:** ^1^ Department of Microbiology, Vardhman Mahavir Medical College and Safdarjung Hospital, New Delhi, India; ^2^ University School of Medicine and Paramedical Health Sciences, Guru Gobind Singh Indraprastha University, Dwarka, India

**Keywords:** *Acinetobacter calcoaceticus–baumannii* (ACB) complex, carbapenem-resistant *Acinetobacter baumannii* (CRAB), loop-mediated isothermal amplification (LAMP) assay, internal transcribing spacer (ITS) 16S–23S rRNA, polymerase chain reaction (PCR), limit of detection (LOD)

## Abstract

**Background:** *Acinetobacter calcoaceticus–baumannii* (ACB) complex has emerged as an important nosocomial pathogen and is associated with life-threatening infections, especially among ICU patients, including neonates. Carbapenem resistance in *Acinetobacter baumannii* has emerged globally and is commonly mediated by *bla*_OXA-23_. Clinically significant infections with carbapenem-resistant *Acinetobacter baumannii* (CRAB) are a major concern since therapeutic options are limited and associated mortality is high. Early diagnosis of both the pathogen and resistance is important to initiate the optimal therapy and prevent selection of resistance. In the current study, a loop-mediated isothermal amplification (LAMP) assay was developed for rapid detection of the ACB complex and carbapenem resistance mediated by *bla*_OXA-23_.

**Methodology:** Universal LAMP primers were designed for the detection of significant members of the ACB complex and carbapenem resistance targeting the ITS 16S–23S rRNA and *bla*_OXA-23_ gene respectively. The optimal conditions for the LAMP assay were standardized for each primer set using standard ATCC strains. The sensitivity of the LAMP assay was assessed based on the limit of detection (LOD) using different DNA concentrations and colony counts. The specificity of LAMP was determined using the non-ACB complex and non-*Acinetobacter species*. The results of the LAMP assay were compared with those of polymerase chain reaction (PCR).

**Results:** The optimal temperature for the LAMP assay was 65°C, and the detection time varied with various primers designed. Using the ITS Ab1 primer, LODs of LAMP and PCR assays were 100 pg/μl and 1 ng/μl of DNA concentration and 10^4^ cfu/ml and 10^8^ cfu/ml of colony count, respectively. The LAMP assay was 10- and 10^4^-fold more sensitive than PCR using DNA concentration and colony count, respectively. The LAMP assay was found to be specific for clinically important ACB complex species.

**Significance of the study:** The LAMP assay can be applied for early detection of significant species of the ACB complex from clinical samples and their carbapenem-resistant variants. Depending on the emerging pathogen and locally prevalent resistance genes, the LAMP assay can be modified for detection of colonization or infection by various resistant bugs.

## Introduction

*Acinetobacter species* are a group of aerobic, non-fermentative, oxidase-negative, non-motile, gram-negative coccobacilli, and they have emerged as important opportunistic pathogens in healthcare facilities (Peleg et al., 2008; Howard et al., 2012; Akrami and Namvar, 2019). The *Acinetobacter calcoaceticus–baumannii* (ACB) complex is most commonly associated with life-threatening infections in humans such as sepsis and ventilator-associated pneumonia (VAP). The ACB complex comprises four different members: *Acinetobacter baumannii* (*A. baumannii*), *Acinetobacter pittii* (*A. pittii*; genomospecies 3), *Acinetobacter nosocomialis* (*A. nosocomialis*; genomospecies 13TU), and *Acinetobacter calcoaceticus* (*A. calcoaceticus*) (Prashanth and Badrinath, 2006; Chusri et al., 2014).

Within the ACB complex, *A. baumannii* has emerged as an important pathogen associated with a variety of nosocomial infections, including bacteremia, pneumonia, meningitis, and urinary tract and surgical site infections (Manchanda et al., 2010; Chen et al., 2014). *A. baumannii* has rapidly acquired resistance to multiple antibiotics, and multidrug resistance (MDR) is common. Recently, studies have reported that the infection rates of *A. pittii* and *A. nosocomialis* have been gradually increasing among hospital patients (Nemec et al., 2011; Wang X et al., 2013; Chusri et al., 2014).

Carbapenem resistance in MDR *A. baumannii* has emerged globally, and infections with carbapenem-resistant *Acinetobacter baumannii* (CRAB) are of major concern as limited therapeutic options are available for management and they are associated with high mortality (Lemos et al., 2014; Butler et al., 2019). The reported mortality rate among patients with sepsis caused by *A*. *baumannii* is up to 20% in neonates and varies from 16.3% (outside ICU) to 43.4% (within ICU) among adults (Peleg et al., 2008; De et al., 2013; Nazir, 2019). The β-lactam resistance in *A. baumannii* is mediated by various β-lactam–hydrolyzing enzymes, i.e., class A (extended-spectrum β-lactamases, ESBLs), class B (metallo-β-lactamases, MBLs), class C (ampicillinase C, AmpC), and class D (OXA type). The subgroups of carbapenem-hydrolyzing OXA’s enzymes prevalent in *A. baumannii* are OXA-23, OXA-24, OXA-51, and OXA-58 (Butler et al., 2019). However, the *bla*_OXA-23_ gene has been disseminated worldwide, and the frequency of OXA-23 producing *A. baumannii* strains in clinical settings is significantly high (Hsu et al., 2017; Butler et al., 2019; Jain et al., 2019; Kumar et al., 2019; Vijayakumar et al., 2019). These enzymes are primarily encoded on plasmids and easily transmissible within gram-negative bacteria.

In patients with sepsis, early detection of both the pathogen and associated drug resistance is important for optimizing therapy and preventing selection of resistance. Every 1-h delay in antibiotics may lead to a 3–7% increase in the odds of a poor outcome (Seymour et al., 2017). The turnaround time (TAT), for identification of the pathogen along with its sensitivity profile, is at least 24–48 h using culture-based diagnostic methods (conventional or automated) (Ahmad et al., 2017). In recent years, MALDI-TOF (matrix-assisted laser desorption ionization time-of-flight) mass spectrometry has been established as a diagnostic tool for identification of microorganisms from pure cultures. However, the high initial investment for the equipment and its limited application for detection of drug resistance in routine diagnostic laboratories are some of the major existing challenges (Kostrzewa et al., 2013; Oviaño at al., 2016). Molecular-based techniques, like polymerase chain reaction (PCR), can further reduce the TAT for the final result with better specificity but are not widely used in resource-limited countries as they require considerable skill, expensive equipment, and trained personnel (Drapała and Kordalewska, 2013). A simple, cost-effective, and rapid method is required to detect both the pathogen and associated antibiotic resistance within the shortest TAT in hospitals with limited resources.

In recent years, loop-mediated isothermal amplification (LAMP) is a widely used technique for rapid diagnosis of infectious diseases in hospitals as it offers a field-friendly alternative to PCR (Drapała and Kordalewska, 2013). Previously, the LAMP assay has been widely used for detection of various pathogens, including influenza A subtypes H1N1, H5N1, and H7N9, *Mycoplasma pneumoniae*, *Mycobacterium tuberculosis*, *Neisseria meningitidis*, *Plasmodium vivax*, and human immunodeficiency virus (McKenna et al., 2011; Haleyur et al., 2014; Modak et al., 2016; Yadav et al., 2017; Ahn et al., 2019).

LAMP is a simple, rapid, and specific tool that amplifies the DNA under isothermal conditions using *Bacillus stearothermophilus* (*Bst*) DNA polymerase and a set of four (to six) specifically designed primers. The ease of detection of amplification and the simplicity of the assay are the hallmarks of this technique (Notomi et al., 2002; Abdullahi et al., 2015). In addition, it eliminates the need for expensive equipment or technical expertise. Hence, it can be used as a rapid tool for point-of-care (POC) diagnosis in healthcare facilities particularly in the resource-constrained settings.

Studies for detection of *A. baumannii* using the LAMP assay are scarce and are limited to detection of either *A. baumannii* or carbapenem resistance (Soo et al., 2013; Wang Q et al., 2013; Li et al., 2015; Yamamoto et al., 2015; Garciglia et al., 2020; Khirala et., 2020; Tesalona et al., 2020). In the present study, the LAMP assay was designed for detection of clinically significant members of the ACB complex (*A. baumannii*, *A. pittii*, and *A. nosocomialis*) and associated carbapenem resistance.

## Material and Methods

### Study Settings (Study Site and Ethical Consideration)

**FIGURE 1:**
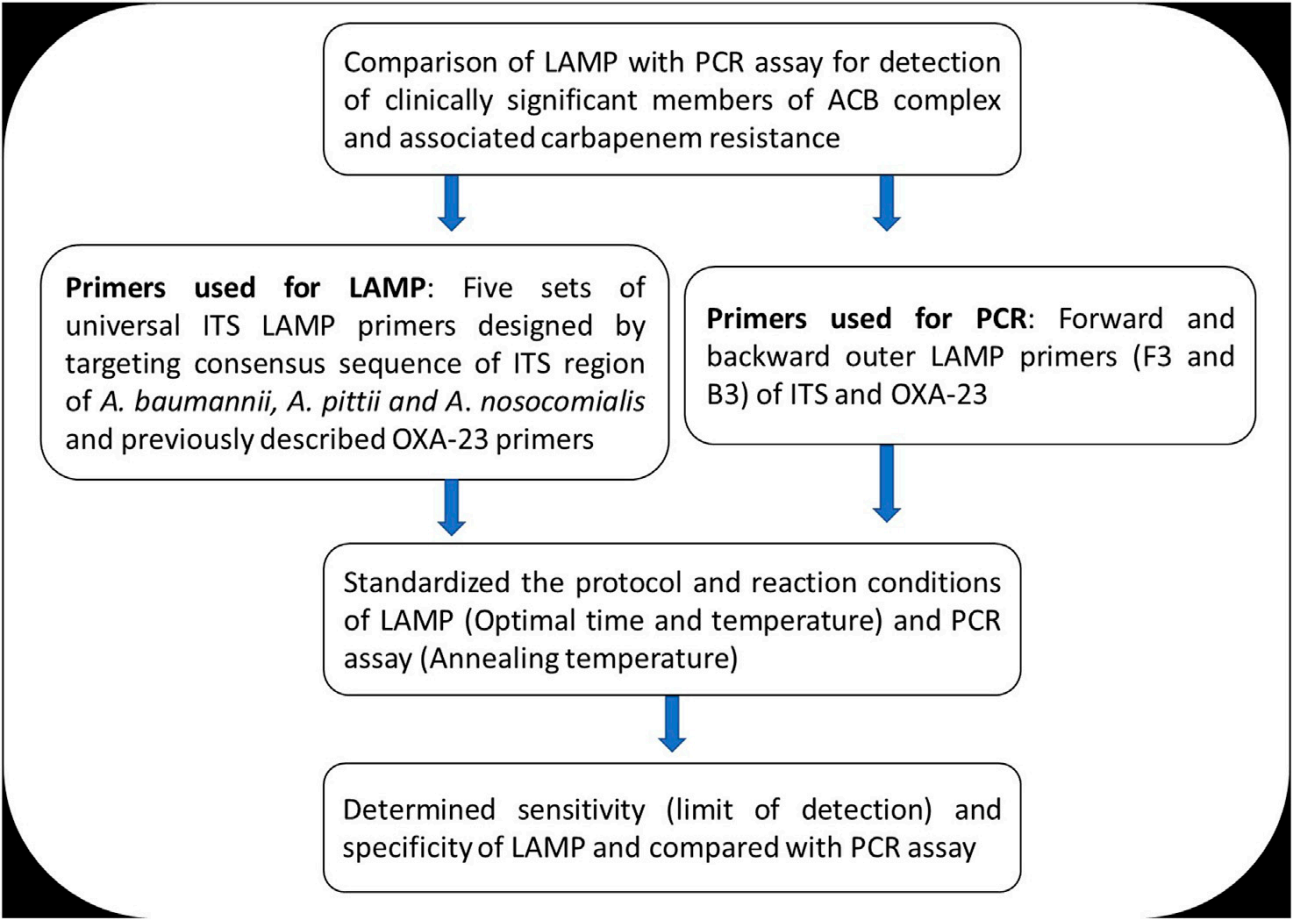
Summary of the study workflow.

The study was conducted at the Department of Microbiology, Vardhman Mahavir Medical College and Safdarjung Hospital, New Delhi, from May 2018 to February 2019. The LAMP assay was standardized for detection of clinically significant members of the *Acinetobacter calcoaceticus–baumannii complex* (*A. baumannii*, *A. pittii*, *and A. nosocomialis*) targeting the internal transcribing spacer (ITS) region between 16S and 23S rRNA genes and associated carbapenem resistance mediated by *bla*_OXA-23_. This study was approved by the institutional ethics committee (IEC/VMMC/SJH/Project/April 2018/1056). The workflow of the study is summarized in Figure 1.

### Design of LAMP Primers

**FIGURE 2:**
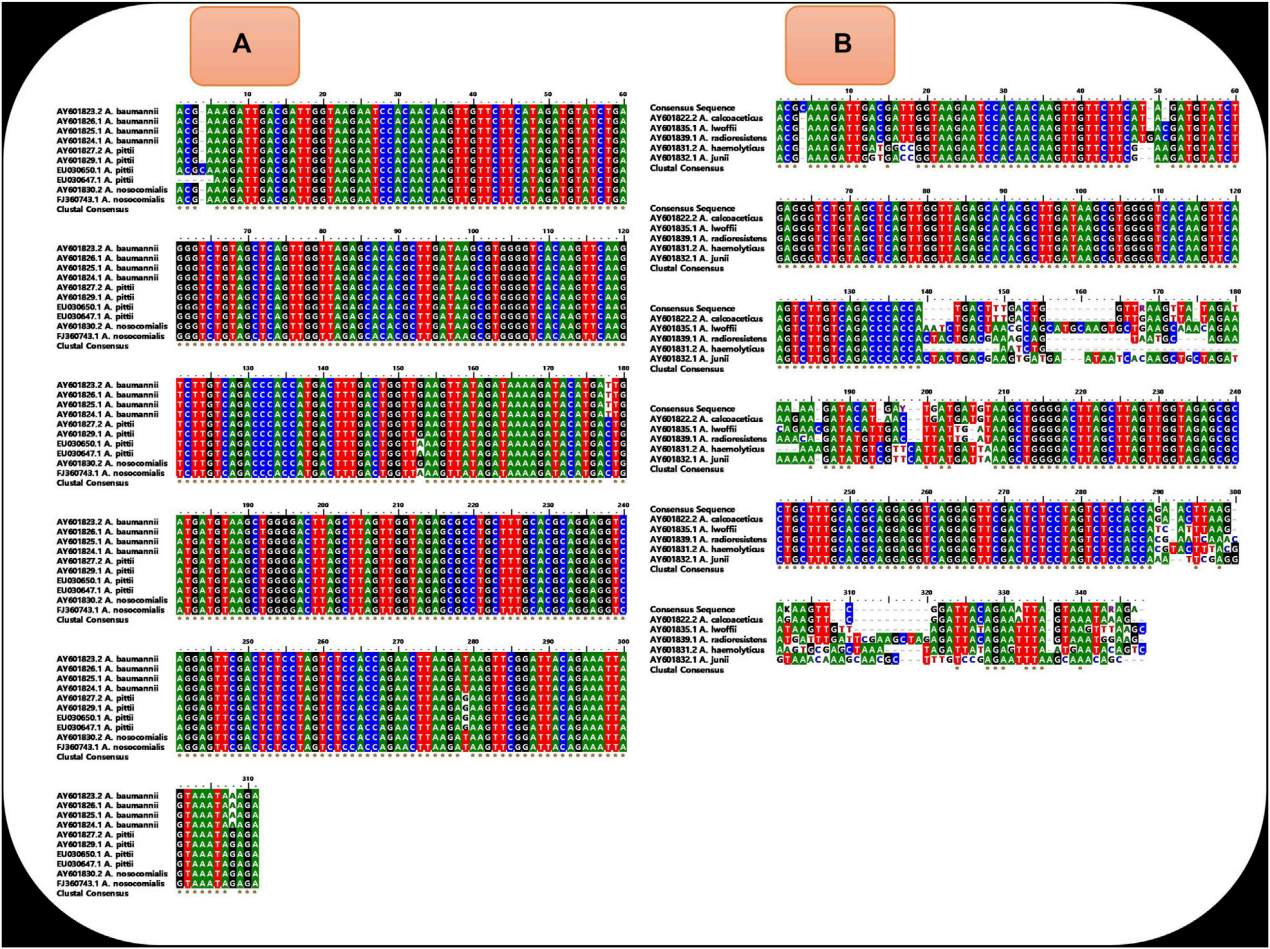
**(A)** shows the alignment of sequence of diverse isolates of *A. baumannii* (*N*=4), *A. pittii* (*N* = 4), and *A. nosocomialis* (N=2) and demonstrates that the sequences are conserved across diverse isolates of three species except few locations. **(B)** shows the alignment of our consensus ITS 16S–23S rRNA sequence with the sequence of other *Acinetobacter* species (*A. calcoaceticus*, *A. lwoffii*, *A. radioresistens*, *A. haemolyticus*, and *A. junii*). The presence of the non-conserved region at locations from 140 to 210 (where our two to three primers are aligned) demonstrates that our LAMP primers were not specific for other *Acinetobacter* species.

**FIGURE 3:**
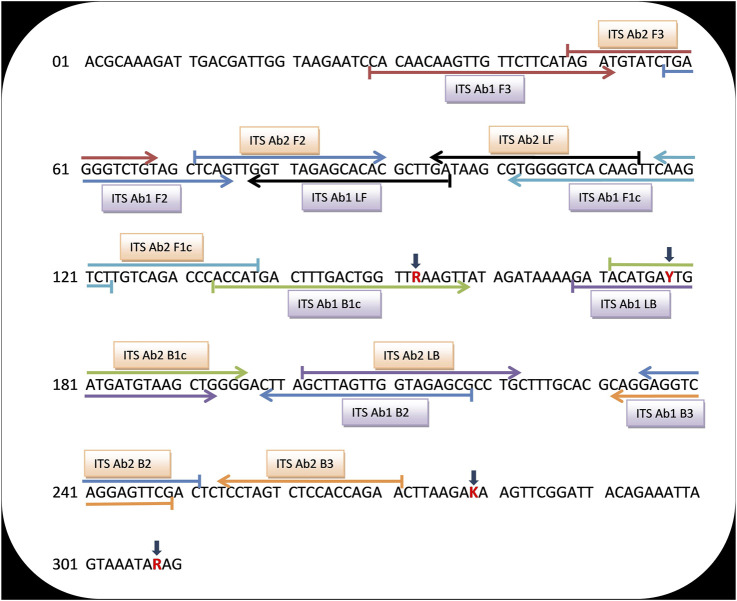
A universal consensus sequence of the ITS 16S–23S rRNA gene that was used to design the LAMP primers for detection of significant members of the *Acinetobacter calcoaceticus–baumannii* (ACB) complex. Red color bases signify the mixed bases used in place of different bases present in *A. baumannii*, *A. nosocomialis*, and *A. pittii*. The sequences sites for ITS Ab1 and Ab2 LAMP primers are designated by upper and lower horizontal arrows, respectively. Right and left arrows indicate sense and reverse complementary sequences that were used.

Universal LAMP primers were designed for detection of *A. baumannii*, *A. pittii*, and *A. nosocomialis* using nucleotide sequences of the ITS region between 16S and 23S rRNA genes of respective strains. A total of 10 sequences (four sequences each from *A. baumannii* and *A. pittii* and two sequences from *A. nosocomialis*) were retrieved from the NCBI database (http://www.ncbi.nlm.nih.gov/) using respective GeneBank accession numbers: AY601823.2, AY601826.1, AY601825.1, AY601824.1 (*A. baumannii*), EU030650.1, EU030647.1, AY601827.2, AY601829.1 (*A. pittii*), and AY601830.2, FJ360743.1 (*A. nosocomialis*). Using the BioEdit Sequence Alignment Editor (Informer Technologies, Inc.), a common sequence was aligned from the conserved location of these 10 sequences (Figure 2A). However, within this common sequence, different nucleotide bases were present in *A. baumannii*, *A. nosocomialis*, and *A. pittii* at position numbers 153 (G,G,A), 178 (T,C,C), 279 (T,T,G), and 308 (A,G,G). Nucleotide bases located at positions 153, 178, 279, and 308 were replaced by mixed bases R, Y, K, and R, respectively, and a consensus sequence was generated (Figure 3).

• Alignment of the above 10 sequences demonstrates that our consensus sequence is conserved across the diverse sequence of three *species* (Figure 2A). Hence, the consensus ITS 16S–23S rRNA sequence shown in Figure 3 was finally used to design the LAMP primers that included forward and backward outer primers (F3 and B3), forward and backward inner primers [FIP(F1c + F2) and BIP(B1c + B2)], and forward and backward loop primers (LF and LB). This consensus sequence was also aligned with the sequence of *Acinetobacter calcoaceticus* and non-ACB complex *species* (*A. lwoffii*, *A. radioresistens*, *A. haemolyticus*, *and A. junii*) to study the specificity of primers (Figure 2B).
• A total of 30 sets of outer and inner LAMP primers were designed using Primer Explorer V5 version software (Eiken Chemical Co., Ltd., Tokyo, Japan). The selection of primers was based on various criteria and included length (18–25 bp), melting temperature (Tm) (60–63°C for F1c and B1c and 55–58°C for F2, B2, F3, and B3), GC content (30–65%), and the distance between primers (120–180 bp for F2 and B2, 45–60 bp for F2 and F1c, 2–20 bp for F3 and F2, and 5–100 bp for F1c and B1c). Five sets of ITS primers were selected based on the stability of either end of each primer (using the dG value ≤−4.0 K cal/mol). However, positions of bases were shifted in order to design the loop primers manually and to fulfill the selection criteria. Finally, all the primer sequences were tested against the Basic Local Alignment Search Tool-National Center for Biotechnology Information (BLAST-NCBI, http://blast.ncbi.nlm.nih.gov/Blast.cgi) to ensure specificity.

The *bla*_OXA-23_-mediated carbapenem resistance was detected using OXA-23 LAMP primers described previously by Yamamoto et al. (2015).

Performance of the LAMP assay was validated with PCR. Forward and backward outer LAMP primers (F3 and B3) of ITS and OXA-23 were used for the PCR assay. The primers used in this study were procured from Integrated DNA Technologies, Inc., United States, in lyophilized form (Table 1). Primers were reconstituted using nuclease-free water and stored at −20°C.

**TABLE 1:** Primers used in the study.

Target gene (primer)	Primer type	Primer sequences (5′–3′)	Reference
16S–23S rRNA Internal Transcribed Spacer (ITS Ab1)	ITS Ab1 F3^a^	CAC​AAC​AAG​TTG​TTC​TTC​ATA​GAT	This study
	ITS Ab1 B3^a^	CGAACTCCTGACCTCCTG	
	ITS Ab1 FIP	AGA​CTT​GAA​CTT​GTG​ACC​CCA​CTG​AGG​GTC​TGT​AGC​TCA​G	
	ITS Ab1 BIP	ACCATGACTTTGACTGGTTRAAGTTCGCTCTACCAACTAAGCTAAG	
	ITS Ab1 LF	TCA​AGC​GTG​TGC​TCT​AAC​C	
	ITS Ab1 LB	GATACATGAYTGATGATGTAAGCTG	
16S–23S rRNA Internal Transcribed Spacer (ITS Ab2)	ITS Ab2 F3^a^	AGA​TGT​ATC​TGA​GGG​TCT​GT	This study
	ITS Ab2 B3^a^	TTCTGGTGGAGACTAGGA	
	ITS Ab2 FIP	ATG​GTG​GGT​CTG​ACA​AGA​CTT​GTC​AGT​TGG​TTA​GAG​CAC​AC	
	ITS Ab2 BIP	ACATGAYTGATGATGTAAGCTGGGGGTCGAACTCCTGACCTC	
	ITS Ab2 LF	GAT​AAG​CGT​GGG​GTC​ACA​AG	
	ITS Ab2 LB	CAG​GCG​CTC​TAC​CAA​CTA​AGC	
*bla*_OXA-23_ (OXA-23)	OXA-23 F3^a^	GAA​GCC​ATG​AAG​CTT​TCT​G	Yamamoto N et al., 2015
	OXA-23 B3^a^	GTA​TGT​GCT​AAT​TGG​GAA​ACA	
	OXA-23 FIP	ACC​GAA​ACC​AAT​ACG​TTT​TAC​TTC​TCA​GTC​CCA​GTC​TAT​CAG​GA	
	OXA-23 BIP	CTG​AAA​TTG​GAC​AGC​AGG​TTG​ACT​CTA​CCT​CTT​GAA​TAG​GCG	
	OXA-23 LF	TTT​TGC​ATG​AGA​TCA​AGA​CCG​A	
	OXA-23 LB	CTG​GTT​GGT​AGG​ACC​ATT​AAA​GGT​T	

F3, forward outer primer; B3, backward outer primer; FIP, forward inner primer; BIP, backward inner primer; LF, loop forward primer; LB, loop backward primer. MP primers are used for amplification of ITS 16S–23S rRNA for identification of clinically significant members of the *Acinetobacter calcoaceticus–baumannii* (ACB) complex and bla_OXA-23_ gene for detection of carbapenem resistance.

a Forward and backward outer primers (F3 and B3) of each set of LAMP primers were used for PCR.

### Selection of Bacterial Strains and DNA Extraction

*A. baumannii* ATCC 19606 and a pre-characterized carbapenem-resistant clinical isolate of *A. baumannii* positive for *bla*_OXA-23_ (CRAB/05/RICU/VMMC-SJH) were used to standardize the LAMP and PCR assays. The specificity of assay was determined using diverse strains *of A. baumannii* (*N* = 30), *A. pittii* (*N* = 6), *A. nosocomialis* (*N* = 2), non-ACB complex *species* (*N* = 21), and non-*Acinetobacter species* (*N* = 10). Details of bacterial strains used in this study are summarized in Table 2.

**TABLE 2:** Details of bacterial strains used in the study.

Details of bacterial strains										
*Non-Acinetobacter species*									No.	Type
*Staphylococcus aureus* ATCC 25923									1	Reference
*Enterococcus faecalis* ATCC 29212									1	
*Escherichia coli* ATCC 25922									1	
*Klebsiella pneumoniae* ATCC BAA 1705									1	
*Enterobacter cloacae* ATCC 700323									1	
*Candida krusei* ATCC 6258									1	
*Pseudomonas aeruginosa* ATCC 27858									1	
*Serratia marcescens*									1	Clinical
*Providencia stuartii*									1	
*Stenotrophomonas maltophilia*									1	

*Acinetobacter species*										
ACB complex	*Acinetobacter baumannii* ATCC 19606								1	Reference
	*Acinetobacter pittii* ^a^								6	Clinical
	*Acinetobacter nosocomialis* ^b^								2	
	Carbapenem-resistant *A. baumannii* (CRAB/05/RICU/VMMC-SJH); a pre-characterized clinical isolate positive for *bla*_OXA-23_								1	
	Thirty diverse genotypes of *Acinetobacter baumannii*									
	Pulsotypes	Genotypic characterization			Phenotypic characterization					
		*bla*_OXA-51_	*bla*_OXA-23_	*bla*_NDM-1_	Biofilm production	AST results				
						MER	IMP	NET		
	I	+	+	+	++	R	R	R	1	
	II	+	+	+	++	R	R	I	1	
	III	+	+	+	+	R	R	R	1	
	IV	+	+	−	+++	R	R	R	1	
	V	+	+	−	+++	I	I	S	1	
	VI	+	+	−	++	R	R	R	5	
	VII	+	+	−	++	R	I	R	1	
	VIII	+	+	−	+	R	R	S	3	
	IX	+	+	−	+	R	R	R	11	
	X	+	−	+	+	R	R	R	1	
	XI	+	−	+	−	R	R	S	1	
	XII	+	−	−	+	S	S	S	2	
	XIII	+	−	−	+	R	R	R	1	
Non-ACB complex	*Acinetobacter lwoffii*								6	Clinical
	*Acinetobacter variabilis*								3	
	*Acinetobacter ursingii*								2	
	*Acinetobacter radioresistens*								2	
	*Acinetobacter junii*								2	
	*Acinetobacter schindleri*								2	
	*Acinetobacter indicus*								2	
	*Acinetobacter haemolyticus*								1	
	*Acinetobacter bereziniae*								1	

a Two strains with distinct MLST patterns and four from patients from diverse geographical areas in India.

b Clinical strains isolated in 2018 and 2021; ATCC, American Type Culture Collection; ACB complex, *Acinetobacter calcoaceticus–baumannii* complex; AST, antimicrobial sensitivity testing; MER, meropenem; IMP, imipenem; NET, netilmicin; R, resistant; S, sensitive; I, intermediate. In the genotypic characterization section, + and – are indicative of the presence and absence of gene, respectively. In the biofilm production section: −, +, ++, and +++ are indicative of none, weak, moderate, and strong biofilm producer, respectively.

All reference and clinical strains were sub-cultured on Mueller Hinton Agar (MHA) [HiMedia Pvt. Ltd., India] and incubated at 37°C. After overnight incubation, genomic DNA was extracted using the boiling prep method described previously (Li et al., 2015) with modifications. Briefly, two or three isolated colonies of organisms were suspended in 100 µl of nuclease-free water and mixed well. Bacterial suspension was heated at 100°C for 15 min and then placed on ice for 10 min. Finally, suspension was centrifuged at 12,000 rpm for 5 min. Approximately 60 µl of the supernatant was stored at −20°C for LAMP and PCR assays.

### Controls for LAMP and PCR Assays

The positive control (PC) and negative control (NC) used for the LAMP and PCR assays included *A. baumannii* ATCC 19606 and *Escherichia coli* (*E. coli*) ATCC 25922 for ITS primers and CRAB/05/RICU/VMMC-SJH and *A. baumannii* ATCC 19606 for OXA-23 primers, respectively. For all the reactions, nuclease-free water (NFW) was used as a reagent control (RC).

### Determination of Detection Time for LAMP Assay

The LAMP assay was performed using 25 μl of reaction mixture that included 23 μl of master mix and 2 μl of genomic DNA of control strain or nuclease-free water.

#### Preparation of Master Mix

The master mix for the LAMP assay included 2X of reaction mixture buffer (40 mM HCl (pH 8.8), 20 mM (NH4)_2_SO_4_, 20 Mm KCl, 4 mM MgSO_4_, 0.2% Triton-X), 1.4 mM of each dNTP, 4 mM MgSO_4_, 8 U of *Bst* DNA polymerase (New England Biolabs, United States), 0.8 μM of FIP and BIP, 0.4 μM of LF and LB, and 0.1 μM of F3 and B3.

#### Addition of Genomic DNA and SYBR Green

After preparation of master mix, 2 μl of genomic DNA (about 100 ng/μl concentration) of PC (*A. baumannii* ATCC 19606 for ITS primers and CRAB/05/RICU/VMMC-SJH for OXA-23 primers) and NC (*E. coli* ATCC 25922 for ITS primers and *A. baumannii* ATCC 19606 for OXA-23 primers) and 2 μl of NFW as an RC were added in the respective tubes. For the final step, 2 μl of 1:10 diluted 10,000X of SYBR Green I (Invitrogen, United States) was added to the inner side of the cap of each PCR tube for visual detection of fluorescence.

#### LAMP Conditions and Detection Time

As the activity of *Bst* polymerase enzyme is optimal at temperature 65°C (Chander et al., 2014), the LAMP assay was initially performed at this temperature. The test was performed separately for ITS and OXA-23 primers using multiple sets of PC, NC, and RC (each set for a particular time) in a heating block at 65°C. This included 11 sets for ITS and five sets for OXA-23. To detect the minimum time required for the positive assay, the results were observed every 5 min from 10 to 60 min for ITS primers and from 10 to 30 min for OXA-23 primers. At an interval of every 5 min, a set of PC, NC, and RC were picked and mixed well with the fluorescent dye on the inner side of the cap and the results were observed visually.

#### Result Interpretation

The color of the LAMP product was observed under UV light or by direct visual inspection against a white or black background. The shortest time of detection was also observed. A change in color from orange to green and no color change were indicative of a positive and a negative result, respectively.

### PCR Assay

The PCR was performed using forward and backward outer ITS and OXA-23 LAMP primers (F3 and B3). The annealing temperature of each set was determined using gradient PCR (Eppendorf master cycler, Hamburg, Germany).

The final volume for the PCR reaction mixture was 25 μl that included 23 μl of master mix and 2 μl of genomic DNA of control strains or nuclease-free water. The final reaction mix consisted of following reagents (final concentration): 10 mM Tris-HCl (pH 8.8), 50 mM KCl, 2 mM MgCl_2_, 200 µM of each dNTP, 0.2 µM concentration of each primer (F3 and B3), and 1 U of *Taq* DNA polymerase (Thermo Scientific, United States). After preparation of master mix, 2 μl of genomic DNA (about 100 ng/μl concentration) of PC (*A. baumannii* ATCC 19606 for ITS primers and CRAB/05/RICU/VMMC-SJH for OXA-23 primers) and NC (*E. coli* ATCC 25922 for ITS primers and *A. baumannii* ATCC 19606 for OXA-23 primers) and 2 μl of NFW (as an RC) were added in respective tubes. The PCR assay was performed for 30 cycles after initial denaturation for 5 min at 94°C and each cycle consisting of 30 s of denaturation at 94°C, 30 s of annealing at the determined optimum temperature, and 45 s of extension at 72°C. A final 10 min extension step was performed at 72°C.

The amplified products were analyzed by gel electrophoresis using 1% agarose gel stained with ethidium bromide. A PCR ruler (100 bp) (Thermo Scientific, United States) was used as the molecular-size marker, and Gel images were acquired using a Gel Doc Alpha Imager “EP” system (Alpha Innotech, CA, United States) for interpretation of PCR results.

### Sensitivity of LAMP and PCR Assays Based on Limit of Detection

The limit of detection (LOD) of LAMP and PCR assays was determined in terms of DNA concentration and colony count (cfu/ml) using *A. baumannii* ATCC 19606 (for ITS Ab1 and Ab2 primers) and CRAB/05/RICU/VMMC-SJH (for OXA-23 primers). To determine the LOD using DNA, the concentrations were measured using a Nanodrop 2000c Spectrophotometer (Thermo Scientific, United States) and adjusted to 100 ng/μl. The DNA was then diluted serially (10-fold) using nuclease-free water to produce concentrations ranging from 100 ng/μl to 0.1 fg/μl.

In addition, the LOD was also determined using colony count. Bacterial suspension of 0.5 McFarland standards (≈1.5 × 10^8^ cfu/ml) was prepared from respective strains using a DEN-1 McFarland Densitometer (Biosan Sia, Latvia). The turbidity of suspension was adjusted to 1.0 × 10^8^ cfu/ml and diluted serially (10-fold) to obtain dilutions ranging from 1.0 × 10^8^ to 1.0 × 10^2^ (in cfu/ml). The DNA was extracted from serially diluted suspensions using the boiling prep method.

The LAMP and PCR assays were carried out using 2 μl of genomic DNA obtained from various dilutions of DNA and colony counts along with the NC and RC. The LAMP assay was performed in a heating block at 65°C for 10–60 min (ITS primers) and 10–30 min (OXA-23 primers), and the results were observed at 5 min intervals. For PCR, the test was performed as per conditions specified in *PCR Assay*.

### Effect of Various Temperatures on LAMP Reaction

The limit of detection of LAMP assay was studied at 65°C. The effect of temperature was assessed using least concentration (of DNA and colony count). Duplicate vials of PC, NC, and RC were prepared, and the LAMP assay was performed using the ITS primer at various temperatures ranging from 60 to 65°C (at an interval of 1°C).

### Specificity of LAMP Assay

The specificity of LAMP assay for detection of the ACB complex was studied using 38 diverse strains of significant members of the ACB complex [*A. baumannii* (30), *A. pittii* (6), and *A. nosocomialis* (2)]*.* Thirty *A. baumannii* isolates belonged to 13 pulsotypes, and the phenotypic and genotypic characteristics of these isolates are shown in Table 2. The specificity of LAMP assay was also determined using 21 non-ACB complex species [*A. lwoffii* (6), *A. variabilis* (3), *A. ursingii* (2), *A. radioresistens* (2), *A. junii* (2), *A. schindleri* (2), *A. indicus* (2), *A. haemolyticus* (1), and *A. bereziniae* (1)] and 10 non-*Acinetobacter species* [seven reference strains (*Staphylococcus aureus* ATCC 25923, *Enterococcus faecalis* ATCC 29212, *Escherichia coli* ATCC 25922, *Klebsiella pneumoniae* ATCC BAA 1705, *Enterobacter cloacae* ATCC 700323, *Candida krusei* ATCC 6258, and *Pseudomonas aeruginosa* ATCC 27858) and three clinical isolates (*Serratia marcescens*, *Providencia stuartii*, and *Stenotrophomonas maltophilia*)]. Genomic DNA was prepared from respective strains using the boiling prep method, and LAMP and PCR assays were performed.

## Results

### Selection of Primers

Among the five sets of ITS LAMP primers designed for identification of the consensus region of ITS 16S–23S rRNA gene of *A. baumannii*, *A. pittii*, *and A. nosocomialis*, only two sets of primers (namely, ITS Ab1 and ITS Ab2) were selected based on the BLAST analysis. Both primer sets showed green fluorescence indicating a successful amplification. Primer sequences of ITS Ab1 and ITS Ab2 are indicated by upper and lower arrows, respectively, in Figure 3.

### Optimal Detection Time of LAMP Assay

The LAMP assay was performed at 65°C using 100 ng/μl genomic DNA of the positive and negative controls, and the detection time was 20, 45, and 10 min for the ITS Ab1, Ab2, and OXA-23 primer set, respectively (Table 3). Using SYBR green, the color change from orange to green was indicative of a successful amplification/positive test, whereas no color change was indicative of no amplification/negative test. Among ITS primers, the ITS Ab1 primer set amplified the target sequence within the shortest time.

**TABLE 3:** Limit of detection of LAMP and PCR assays from different DNA concentrations and colony counts using ITS (Ab1 and Ab2) and OXA-23 primers for detection of clinically significant members of the ACB complex and carbapenem resistance, respectively.

Primers	Limit of detection of LAMP and PCR assays			
	DNA concentration (/µl)		Colony count (cfu/ml)	
	LOD of LAMP with detection time	LOD of PCR	LOD of LAMP with detection time	LOD of PCR
ITS Ab1	100 ng (20 min)	1 ng	10^8^ (20 min)	10^8^
			10^7^ (25 min)	
	10 ng (20 min)		10^6^ (25 min)	
	1 ng (25 min)		10^5^ (30 min)	
	100 pg (30 min)		10^4^ (30 min)	
	≤10 pg (ND till 45 min)		≤10^3^ (ND till 45 min)	
ITS Ab2	100 ng (45 min)	1 ng	10^8^ (50 min)	10^8^
	10 ng (50 min)			
	1 ng (50 min)		≤10^7^ (ND till 60 min)	
	100 pg (50 min)			
	≤10 pg (ND till 60 min)			
OXA-23	100 ng (10 min)	100 pg	10^8^ (10 min)	10^8^
	10 ng (10 min)		10^7^ (10 min)	
	1 ng (10 min)		10^6^ (15 min)	
	100 pg (15 min)		10^5^ (15 min)	
	10 pg (15 min)		10^4^ (15 min)	
	1 pg (15 min)		10^3^ (15 min)	
	≤100 fg (ND till 30 min)		≤10^2^ (ND till 30 min)	

ACB complex, *Acinetobacter calcoaceticus–baumannii* complex; LOD, limit of detection; min, minutes; ND, not detected; cfu/ml, colony-forming unit/ml.

### PCR Results

**FIGURE 4:**
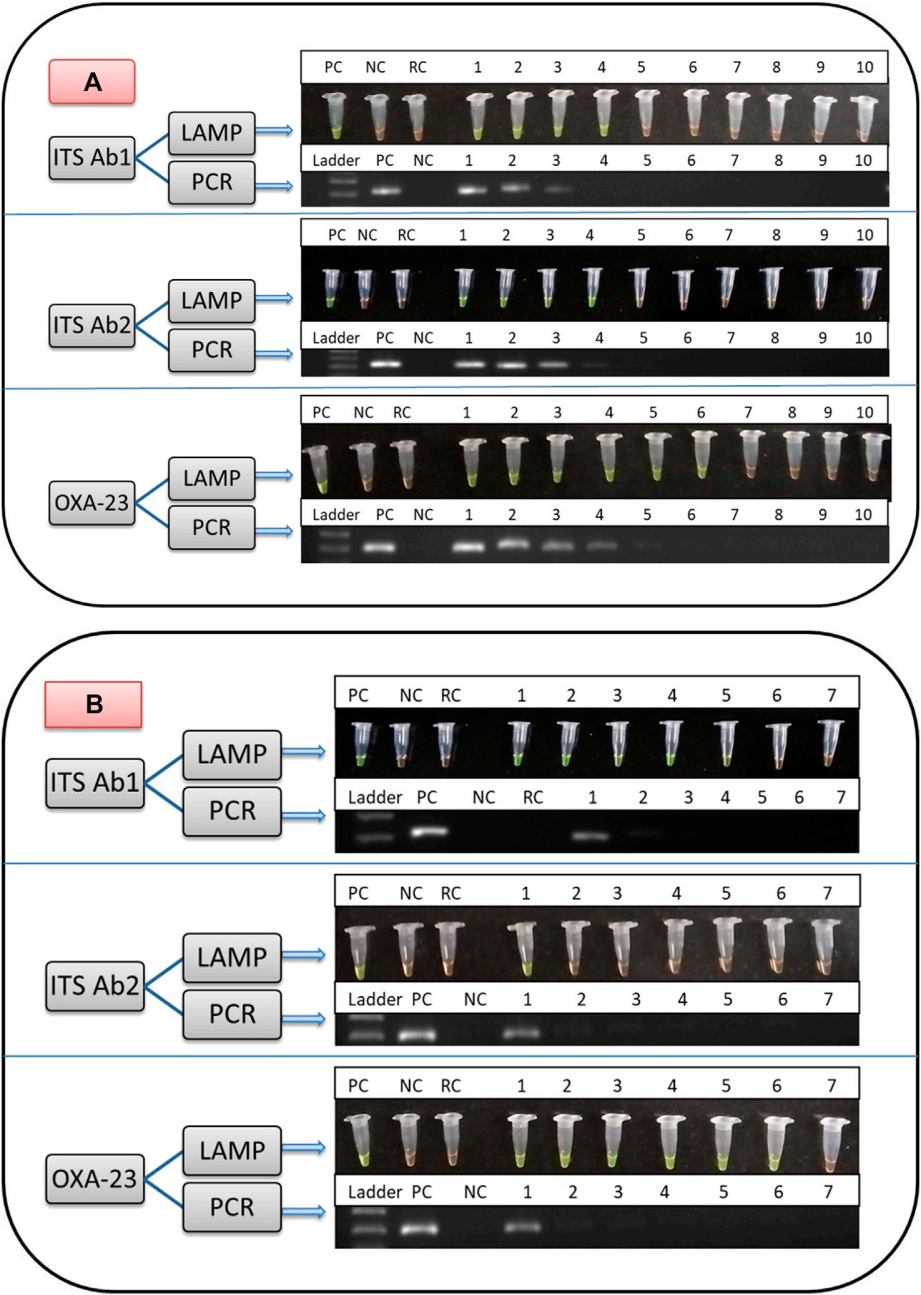
Comparison of the limit of detection (LOD) of LAMP and PCR assays from direct culture for identification of significant members of the ACB complex using ITS Ab1 and Ab2 primers and detection of carbapenem resistance using the OXA-23 primer. **(A)** shows the LOD using different DNA concentrations. Lane for LAMP and PCR: PC, positive control; NC, negative control; RC, reagent control; DNA concentration (/μl) from Lanes 1 to 10; 100, 10, 1 ng, 100, 10, 1 pg, 100 fg, 10 fg, 1 fg, and 0.1 fg, respectively. (B) shows the LOD using different colony counts. Lane for LAMP and PCR: PC, positive control; NC, negative control; RC, reagent control; colony count (cfu/ml) from Lanes 1 to 7: 1.0 × 108, 107, 106, 105, 104, 103, and 102, respectively. In **(A**,**B)**, in the LAMP assay, green and orange colors are indicative of positive and negative results, respectively, and in the PCR assay, the presence and absence of bands are indicative of positive and negative results, respectively.

The optimal annealing temperature for outer LAMP primers (F3 and B3) of ITS Ab1, ITS Ab2, and OXA-23 was 52.5, 52.5, and 50.5°C, respectively. The amplified products from positive control (*A. baumannii* ATCC 19606 for ITS Ab1 and Ab2 and CRAB/05/RICU/VMMC-SJH for the OXA-23 primer) were observed on 1% gel corresponding to a band size of 221, 223, and 200 bp, respectively (Figures 4A,B), while no bands were observed for negative (*E. coli* ATCC 25922 for ITS Ab1 and Ab2 and *A. baumannii* ATCC 19606 for OXA-23) and reagent controls.

### Sensitivity of LAMP and PCR Assays

The limit of detection of LAMP and PCR assays is shown in Figures 4A and B and Table 3. In brief, the detection limit of LAMP assay using ITS Ab1 and Ab2 LAMP primers was 100 pg/μl (using DNA concentration), which was 10-fold more sensitive than PCR (1 ng/μl). While using different colony counts, the detection limit of LAMP for ITS Ab1 and Ab2 primers was 1 × 10^4^ and 1 × 10^8^ cfu/ml, respectively. Using ITS Ab1, the LAMP assay was 10^4^-fold more sensitive than PCR (1 × 10^8^ cfu/ml). Furthermore, the detection limit of LAMP using the OXA-23 primer was 1 pg/μl (using DNA concentration) and 1 × 10^3^ cfu/ml (using colony counts), which was 10^2^-fold and 10^5^-fold more sensitive than PCR (100 pg/μl and 1 × 10^8^ cfu/ml, respectively). The detection time of LAMP assay using ITS Ab1, ITS Ab2, and OXA-23 primers was 20, 45, and 10 min, respectively, for the highest concentration and 30, 50, and 15 min for the lowest concentration (Table 3).

• The LAMP assay was performed at various temperatures ranging from 60 to 65°C. A complete change in color from orange to green was observed at 63–65°C. In contrast at 60–62°C, this color change was not visually distinct. Hence, the optimal temperature range for the LAMP reaction was 63–65°C (Supplementary Figure S1).

### Specificity of LAMP

The specificity of the LAMP assay was determined using ACB complex (*A. baumannii*, *A. pittii*, and *A. nosocomialis*), non-ACB complex, and non-*Acinetobacter species*. PCR detected both ACB complex *species* (*N* = 38) and non-ACB complex *species* (*N* = 21) and was negative for all non-*Acinetobacter species* (*N* = 10). In contrast, the LAMP assay was more specific as all diverse strains of *A. baumannii*, *A. pittii*, and *A. nosocomialis* were amplified successfully, while non-ACB complex and non-*Acinetobacter species* were not amplified (Supplementary Figure S2).

## Discussion

In the last decade, the reports of infections associated with the ACB complex among hospitalized patients have increased. *A. baumannii* is emerging as an important cause of pneumonia, bacteremia, urinary tract infection, meningitis, and wound infections (Manchanda et al., 2010; Chen et al., 2014). Various countries, including India, have reported high resistance to carbapenems in *A. baumannii*, ranging from 40 to 75% (Vijayakumar et al., 2019). CRAB has been classified as a priority one pathogen among the 12 antibiotic-resistant “priority pathogens” by the WHO (Tacconelli et al., 2018). Resistance to carbapenems is mediated by various OXA enzymes (OXA-23, OXA-51, OXA-58, and OXA-24) (Hsu et al., 2017; Butler et al., 2019).

*Acinetobacter* harboring *bla*_OXA-23_ have emerged globally and are also the most prevalent carbapenemase gene circulating in Asian countries, particularly in China and India (Mugnier et al., 2010; Liu et al., 2015; Jain et al., 2019; Kumar et al., 2019; Vijayakumar et al., 2019). In a multicentric study (Vijayakumar et al., 2019) reported from India, *bla*_OXA-23-like_ was the most common mechanism of carbapenem resistance and was observed in 97% of isolates. In this study, carbapenem resistance mediated by *bla*_OXA-24-like_ and *bla*_OXA-58-like_ was not detected. Similarly, Kumar et al. (2019) observed a high prevalence of *bla*_OXA-23-like_ (97.7%) in CRAB followed by *bla*_NDM-1_ (29.1%) and *bla*_OXA-58-like_ (3.5%). In a recent study among patients with VAP from our center (Jain et al., 2019), resistance was mediated by *bla*_OXA-23-like_ in 96.3% (26/27) of CRAB. These studies suggest that *bla*_OXA-23-like_ can be used as a screening marker for detection of carbapenem resistance among *Acinetobacter* isolates and used in this study.

Early diagnosis of clinically significant ACB complex infections, associated with carbapenem resistance, is important to initiate early optimal therapy, implement antibiotic stewardship programs, and prevent selection of resistance. Conventional culture-based detection methods are time-consuming and delay the optimum therapy, resulting in high morbidity and poor outcomes (Ahmad et al., 2017). Other molecular-based diagnostic methods require initial investments, maintenance, and proprietary ownership, and thus, these techniques are not a suitable option for resource-limited countries (Drapała and Kordalewska, 2013). A point-of-care (POC) test is required for identification of the pathogen and associated drug resistance for optimal management of life-threatening infections, screening of patients colonized with resistant strains, and preventing selection and dissemination of resistant isolates.

LAMP is a qualitative assay, in which the DNA is amplified under isothermal conditions and the results are interpreted visually by appearance of turbidity or fluorescence. Low investment, easy interpretation, simplicity, and rapid detection are the most significant features of the LAMP assay, which make it a useful POC test (Abdullahi et al., 2015; Notomi et al., 2002).

• The strength of the study was that the LAMP assay was standardized for detection of the other emerging clinically important members of the ACB complex, i.e., *A. pittii* and *A. nosocomialis* in addition to *A. baumannii*, and associated carbapenem resistance. In addition, two targets, namely, ITS 16S–23S rRNA and *bla*_OXA-23_, were used for identification of significant members of the ACB complex and carbapenem resistance, respectively. Previous studies have designed the LAMP assay for identification of *A. baumannii* using a single target. The various targets used in these studies are *bla*_OXA-51_ (Li et al., 2015; Garciglia et al., 2020), ITS 16S–23S rRNA (Soo et al., 2013), adeS (Khirala et., 2020), and *bla*_OXA-23_ (Yamamoto et al., 2015 and Tesalona et al., 2020; Garciglia et al., 2020)*.* The application of these assays is limited as they detect either the pathogen or associated drug resistance. The assay using *bla*_OXA-51_, ITS 16S–23S rRNA, and adeS as targets only detects *A. baumannii* rather than significant members of the ACB complex, while that using *bla*_OXA-23_ only detects carbapenem-resistant *A. baumannii.* The other strength of this assay was that the LOD and sensitivity of the assay were detected using both DNA concentration (/µl) and colony count (cfu/ml).

Various primers were designed targeting the ITS 16S–23S rRNA region for identification of *A. baumannii*, *A. pittii*, *and A. nosocomialis*, and two primers (ITS Ab1 and Ab2) were selected for the final assay, which was performed at 65°C, the optimal temperature for *Bst* polymerase (Chander et al., 2014) and carbapenem-resistant gene (OXA-23).

The sensitivity of LAMP assay, based on the LOD, was compared with that of PCR. The LAMP assay was found to be more sensitive in terms of both DNA concentration (/µl) and colony count (cfu/ml).

The majority of studies have validated the LOD of LAMP with PCR using different concentrations of DNA. In the present study, the detection limit of LAMP assay for ITS Ab1 and *bla*_OXA-23_ primers was 100 pg/μl and 1 pg/μl of DNA, respectively, which was 10- and 100-fold more sensitive than PCR (Table 3). The similar findings were observed in previous studies in which LAMP was found to be 100-fold (Soo et al., 2013; Yamamoto et al., 2015) and 10-fold (Li et al., 2015; Garciglia et al., 2020) more sensitive than PCR.

While using different colony counts (cfu/ml), the detection limit of LAMP assay for ITS Ab1 and *bla*_OXA-23_ primers was 1 × 10^4^ and 1 × 10^3^ cfu/ml, respectively, which was 10^4^- and 10^5^-fold more sensitive than PCR (Table 3). Limited studies have reported the LOD of LAMP using colony count. Wang Q et al. (2013) have studied the LOD using different colony counts, and RealAmp (qLAMP) was used for identification of *A. baumannii* targeting the pgaD gene. In this study, the LOD of LAMP was 1 × 10^3^ cfu/ml, which was 10-fold more sensitive than conventional PCR (1 × 10^4^ cfu/ml).

Based on the limit of detection, the detection time of our LAMP assay varied between 20 and 30 min, 45 and 50 min, and 10 and 15 min for ITS Ab1, ITS Ab2, and OXA-23 primers, respectively (Table 3). As the shortest detection time was observed with primers ITS Ab1 and OXA-23, they are recommended as optimal primer sets for the assay. In previous studies, the optimal temperature and detection time were similar to our findings and reported to be 65°C for 60 min (Li et al., 2015; Garciglia et al., 2020), 58–64°C for 30 min (Soo et al., 2013), and 65°C for 20 min (Yamamoto et al., 2015).

• In our study, the LAMP assay was found to be highly specific and was negative for all non-*Acinetobacter species* and non-ACB complex *species*, and these findings were in concordance with those of previous studies conducted by Soo et al. (2013) and Li et al. (2015). On the contrary, all diverse clinical strains of *A. baumannii*, *A. pittii*, and *A. nosocomialis* were detected by using ITS primers, which demonstrates that the LAMP assay can be established for different bacterial clones associated with different genotypes, and the diversity of the strains does not affect the assay. The similar findings were observed by Yamamoto et al. (2015), in which 113 bacterial strains of CRAB with different genotypes were detected by the LAMP assay using *bla*_OXA-23_ as a target.

There were a few limitations of this study. Although this study was designed for detection of clinically significant members of the ACB complex (*A. baumannii*, *A. pittii*, and *A. nosocomialis*), only eight diverse strains of *A. pittii* (6) and *A. nosocomialis* (2) could be included for testing of the assay as infections associated with these species are limited or underreported (Chatterjee et al., 2016). The second limitation of this study is that the assay has to be performed in two steps for identification of both the pathogen and carbapenem resistance. As, in the LAMP assay, four to six primers are required for amplification of one target, if multiple targets are used in a single assay, it causes primer–primer interaction and leads to a false positive result.

Early diagnosis of life-threatening infections (i.e., sepsis, pneumonia, and UTI) and colonization (rectal carriage and environmental screening) with a resistant pathogen is important for optimal management and prevention of colonization and further dissemination of the resistant pathogen. In such conditions, the LAMP assay is a potential promising POC test for detection of resistant pathogens. Before this assay can be used in a diagnostic lab, more experiments need to be performed with direct clinical samples to establish the efficacy of the LAMP assay.

In conclusion, the LAMP assay can be used as a potential (simple, rapid, and cost-effective) tool for rapid detection of pathogens, especially in resource-constrained countries, and can be adapted for detection of pathogens from various clinical samples such as blood and other fluids. Also, depending on the emerging pathogen and locally prevalent resistant gene, the assay can be modified accordingly.

## Contribution

A rapid molecular diagnostic tool has the most important role in the management of pathogens associated with life-threatening infections (i.e., sepsis, pneumonia, and UTI) and colonization (rectal carriage and environmental screening). As per the WHO factsheet, October 13, 2020, antimicrobial resistance (AMR) is one of the top 10 global public health threats faced by humanity. Early detection of resistance is important for optimal management, prevention of colonization, and further dissemination of resistant pathogens. Every 1-h delay in antibiotics may lead to a 3–7% increase in the odds of a poor outcome. Carbapenem-resistant *Acinetobacter baumannii* has been classified as a priority one critical pathogen by the WHO and is of major concern because of the limited therapeutic options and high mortality rate.

Conventional culture-based methods are time-consuming, and molecular-based methods require initial investments, maintenance, and proprietary ownership. The LAMP assay is a simple, rapid, and cost-effective molecular technique that can be used as a potential point of care for rapid detection of emerging resistant pathogens. LAMP is a qualitative assay, in which the DNA amplifies under isothermal conditions and the results can be interpreted easily. Also, depending on the emerging pathogen and locally prevalent resistant gene, the assay can be modified accordingly.

## Data Availability

The original contributions presented in the study are included in the article/Supplementary Material, and further inquiries can be directed to the corresponding author.
