# Supplementary figures and images for: Development of Loop-Mediated Isothermal Amplification Assay for Detection of Clinically Significant Members of Acinetobacter calcoaceticus–baumannii Complex and Associated Carbapenem Resistance
Source: Front Mol Biosci. 2021 Jun 23;8:659256. doi: 10.3389/fmolb.2021.659256 (PMC8260673; doi:10.3389/fmolb.2021.659256)

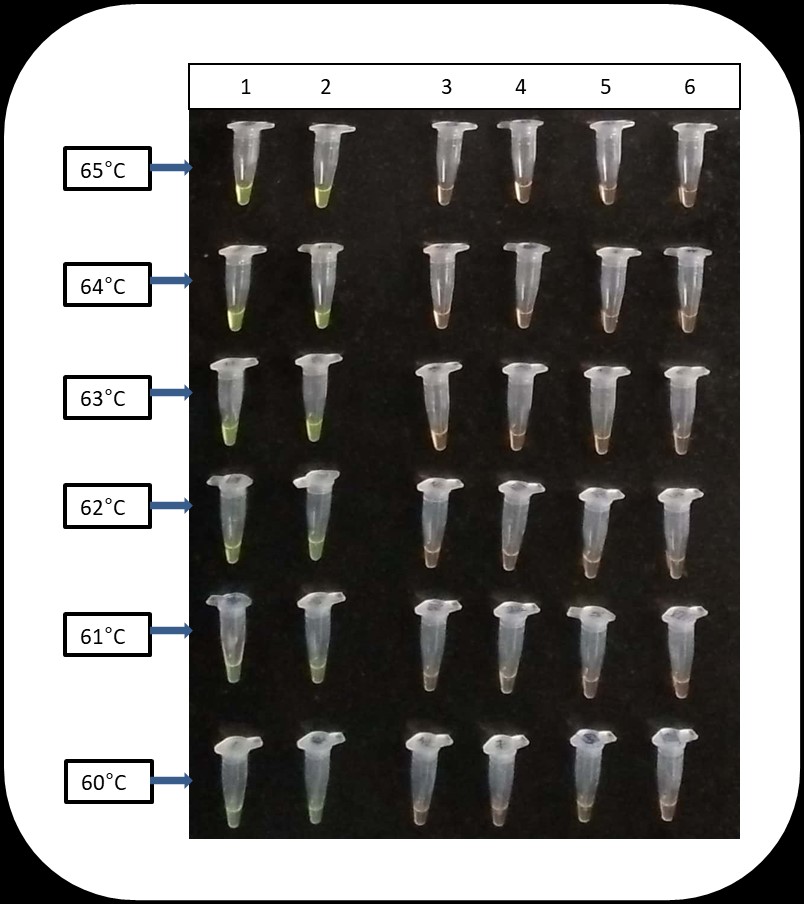

Supplement: Supplementary file 1 [file Image1.JPEG]

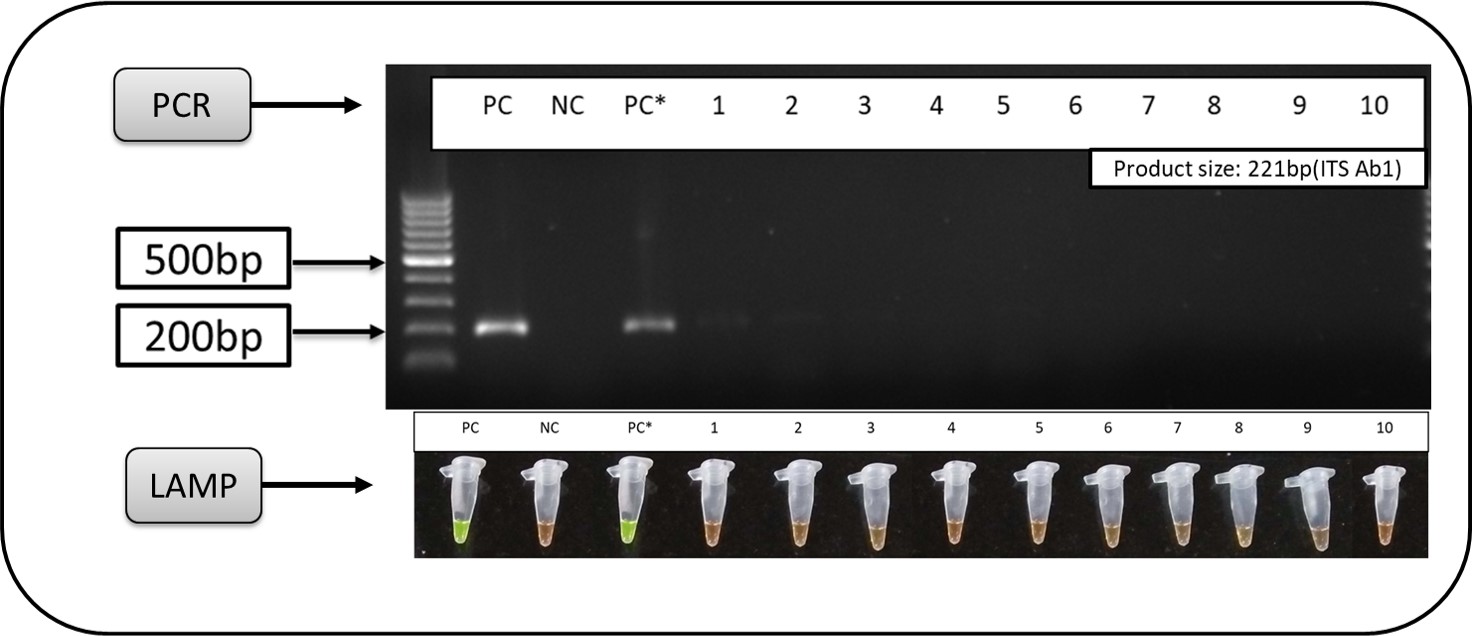

Supplement: Supplementary file 2 [file Image2.JPEG]
